# Permanent Contraception: Women’s Desire for Their Spouse and Future Uptake

**DOI:** 10.1155/ogi/9565232

**Published:** 2026-01-23

**Authors:** Oluwasomidoyin Bello, Raymond Takpe, Olugbenga Saanu

**Affiliations:** ^1^ Department of Obstetrics and Gynaecology, University College Hospital, PMB 5116, Ibadan, Oyo, Nigeria, uch-ibadan.org.ng; ^2^ Department of Obstetrics and Gynaecology, College of Medicine, University of Ibadan, Ibadan, Oyo, Nigeria, ui.edu.ng

**Keywords:** antenatal attendees, future uptake, knowledge, perception, permanent contraception, spousal uptake

## Abstract

**Background and Aims:**

Permanent contraception (PC) is a safe and cost‐effective irreversible method of preventing unwanted pregnancy and reducing maternal morbidity and mortality. Only 0.2% of women use PC in Nigeria. This study aimed at assessing women’s knowledge, their desire for spousal and future uptake of permanent contraception.

**Methods:**

A cross‐sectional survey conducted among 256 antenatal attendees at University College Hospital, Ibadan, Nigeria, using semistructured self‐administered questionnaire. Logistic regression analyses were used to determine factors associated with women’s desire and spousal uptake of PC at 95% confidence interval (CI) with a *p* value of ≤ 0.05.

**Results:**

All were aware of PC and majority (180 [70.3%]) had good knowledge of it. About a quarter (26.5%) of the women wish to use PC once they complete their family size with more than half (52.6%) of them willing to use it after four or more children. Only 28.9% will allow their spouse to have vasectomy. Commonest reasons for PC future uptake are completed family size (88.9%) and economic challenges (45.5%). Those with one living child were less likely to have a good knowledge of PC (AOR = 0.527 and 95% CI = 0.278–0.998) and those with fertility issues were twice more likely to have good knowledge (AOR = 2.373 and 95% CI = 1.030–5.466). Respondent’s perception of PC (*p* < 0.001) and “ever had pregnancy complications” (*p* = 0.014) are associated with their desire for spousal uptake.

**Conclusions:**

There is a high level of awareness and good knowledge of PC; however, just a few will use it or allow their spouse to use it in future. There is a need to strengthen counselling and maternal education on PC with the aim to improve its uptake.

## 1. Introduction

Permanent contraception (PC) is the deliberate use of methods to irreversibly prevent unwanted pregnancy through surgical procedures that obstructs or alters the reproductive pathways, making natural conception highly impossible. It is a highly desired and generally used contraceptive option mainly by women around the world who desire never to become pregnant [1]. Unintended pregnancy is an ill‐timed, unplanned or unwanted or not desired at the time of conception and often related with numerous adverse maternal and child health consequences such as mortality [2, 3]. Male sterilization (vasectomy) and female sterilization (tubal ligation) are current permanent methods of contraception, which often involve surgical intervention, offered to couples who have completed childbearing [1, 4].

Family planning has helped reduce maternal mortality and population growth in developed countries [5]. The contraceptive prevalence rate in Nigeria is low compared with those of some other African countries and developed countries [5, 6]. The low rates of contraceptive use in Nigeria and other developing countries are largely influenced by cultural perceptions and misconceptions about family planning methods, poverty, low levels of female education, as well as the unmet need for family planning from unavailability, and lack of access to family planning services [6–9]. In Nigeria, the level of unmet need for family planning among married women of 19% exceeds the prevalence of contraceptive use (17%) and the demographic and health survey reported a relatively poor knowledge of PC with a prevalence of 0.2% among women of reproductive age groups [8]. The reported knowledge of bilateral tubal ligation and vasectomy was 45.1% and 17.8% as well as 39.7% and 32.6% among the women and men, respectively [8].

Globally, 19% of married couple adopted female sterilization; however, its uptake is far less in Sub‐Saharan Africa with a prevalence of approximately 2.5% among married couple [10, 11]. Modern methods of contraception are mostly used by women with the use by men making up a relatively small subset of contraceptive users. Apart from male condom, vasectomy is the only other safe and effective contraceptive method for men. In spite of the growing evidence on the benefits of engaging men in reproductive health decision‐making, fertility rates, unmet need for family planning remain high and the male contraceptive prevalence rate remain low with PC been the least patronised in many Sub‐Saharan African countries [12, 13]. While there are many influential factors, low contraceptive prevalence has been attributed in part to men’s opposition to or noninvolvement in family planning [12]. Male involvement has historically been depicted as unhelpful by hampering women’s decision‐making on use of family planning, or nonexistent among male partners who are absent altogether due to lack of interest in matters related to reproductive health [14, 15]. However, at the same time, men dominate decision‐making regarding family size and their partner’s use of contraceptive methods in many traditionally patriarchal settings [16]. Women point to their spouses’ resistance to family planning as a significant barrier to uptake and continuation, resulting in decisions to use contraceptive methods covertly or not at all [14, 17]. Conversely, several studies have shown increase uptake and continuation of contraceptives use with spousal’s knowledge and involvement [12, 18].

It is evidence that contraceptive uptake involves a couple, and the partner plays a role in decision making. The male contraception prevalence rate remains low with PC been the least patronised among women despite a high fertility rate of 5.3 and Nigerian women having 0.5 more children that they want on the average [8]. This coupled with the fact that there is dearth of studies in Nigeria assessing women’s perception and desire for their spouse to have PC, which shows a knowledge gap because women’s attitude can influence spousal decision‐making and shape the demand for male‐centred contraceptive options. Inadequate awareness of these perspectives may limit the effectiveness of programs and policies designed to expand contraceptive choices for couples, making it essential to address this gap in order to develop inclusive, equitable family planning initiatives that promote male involvement in reproductive health. It is, therefore, against this background that this study explores women’s knowledge, their desire for spousal and future uptake of PC.

## 2. Materials and Methods

### 2.1. Study Design and Population

This was a health facility based cross‐sectional survey conducted at the antenatal clinic of the University College Hospital (UCH), Ibadan, Nigeria, from March to April 2021. The hospital is a tertiary health facility located in Ibadan, which is the largest city in West Africa, serves as a referral centre for private and public primary and secondary health facilities and provides specialist care to many pregnant women within and beyond South‐West Nigeria. The family planning unit provides services for counselling, contraceptive uptake and follow‐up to an average of about 350 clients monthly while an average of 875 and 110 pregnant women attends antenatal and booking clinic monthly, respectively.

All pregnant women attending antenatal clinic during the study period were the eligible study population. The sample size was estimated using the Leslie Kish sample size formula for a single population proportion at 95% confidence level (CI) and 5% margin of error [19]. The anticipated nonresponse rate was 10%. A total of 256 consenting pregnant women were recruited using a systematic random sampling to enhance representativeness and generalizable and minimize selection bias. The first respondent was selected at random from the first five eligible participants, and subsequently, every 5th eligible woman was enrolled. Women presenting with obstetric emergencies or those who did not consent to participate were excluded. During the data collection, eight women did not consent to participate, resulting in a nonresponse rate of 3.1%.

### 2.2. Data Collection and Analysis

Data were collected using a semistructured self‐administered questionnaire in a quiet and private setting. Trained research assistants were available to provide clarification when needed. To ensure data quality, the research assistants received prior training on questionnaire administration and ethical considerations. Completed questionnaires were reviewed daily for completeness, accuracy and consistency. The questionnaire was developed by the authors using information from the literature. The questionnaire comprised of five sections, which included the sociodemographic characteristics, medical and obstetric history, awareness and knowledge of PC and future uptake of PC as well as their desire for spousal uptake. There was acceptable internal consistency among the variables as Cronbach’s alpha for all the variables was 0.77. The questionnaire was pretested among 20 pregnant women to assess for clarity and understanding of the questions and test for validity.

Data was entered, cleaned and analysed using the Statistical Package for Social Sciences Version 23.0. Descriptive statistics (frequencies, percentages, means, and standard deviations) summarized participant characteristics and responses. Bivariate analyses were performed using chi‐square tests for categorical variables and independent t‐tests for continuous variables. Multivariate logistic regression analyses were used to determine factors associated with the women’s desire and spousal uptake of PC, adjusting for potential confounders. Results are reported as odds ratios (OR) with 95% confidence intervals (CI), and statistical significance was set at *p* < 0.05, two‐sided for all analyses. The question “Have you heard of PC” was used to assess awareness. Awareness was assessed by asking the question with a yes or no response. A total score of more than 50% correct responses of the ten knowledge questions on PC with each question having the same weight was classified as good.

### 2.3. Ethical Considerations

Ethical approval was obtained from Oyo State Research Ethics Review Committee (Ad 13/479/1322), and the study was conducted in accordance with the principles of the Declaration of Helsinki. A written informed consent was obtained from all the respondents before administering the questionnaire after careful explanation of the study objectives was made. Confidentiality and anonymity were ensured as names were not required.

## 3. Results

### 3.1. Respondents’ Knowledge, Perception and Reasons for Future Uptake of PC

The mean age of respondents was 30.2 ± 4.82 years (range: 16–43 years), and majority (71.1%) are 26–35 years. All respondents had heard of PC, 70.3% had a good knowledge while only 38 (26.9%) had good perception of PC. The common sources of information were from the health workers (46.5%) and media (42.6%). Two main reasons for desiring PC in the future are completed family size (88.9%) and economic challenges (45.5%). Meanwhile, 28.9% of the respondents’ desire and will allow their spouse to have PC. More than half (52.6%) of women are willing to have PC after four or more children (Table 1).

**Table 1:** Knowledge, perceptions and reasons for future and spousal uptake of permanent contraception (PC).

Variables	Frequency *n* (%)
Knowledge of PC	
Good	180 (70.3)
Poor	76 (29.7)
Perception	
Good	38 (26.9)
Poor	218 (73.1)
Desire for spousal PC uptake (n = 38)	
Yes	11 (28.9)
No	27 (71.1)
Will spouse agree to have PC?	
Yes	30 (78.9)
No	8 (21.1)
When are you willing to use PC in the future?	
After 3 children	18 (47.4)
After 4 or more children	20 (52.6)
Reasons for PC future uptake^∗^	
Completed desired family size	24 (88.9)
Economic challenges	10 (45.5)
Doctor’s advice based on multiple caesarean/vaginal births	1 (1.5)
Medical/obstetrics complication(s) associated with pregnancy(ies)/birth(s)	1 (1.5)

^∗^Multiple responses, among respondents who expressed willingness to use PC in the future (*n* = 38)”.

### 3.2. Relationship Between Respondents’ Sociodemographics With Their Knowledge on PC.

No association exists between respondents’ sociodemographics with their knowledge on PC (Table 2).

**Table 2:** Association between respondents’ sociodemographics with knowledge on PC.

Variable	Knowledge of PC		Total (%)	*X* ^2^	*p* value
	Good (%)	Poor (%)			
Age (years)					
16–25	27 (15.0)	10 (13.2)	37 (14.5)	0.679	0.712
26–35	129 (71.7)	53 (69.7)	182 (71.1)		
≥ 36	24 (13.3)	13 (17.1)	37 (14.5)		
Marital status					
Married	177 (98.3)	75 (98.7)	252 (98.4)	0.043	0.836
Single/separated/divorced	3 (1.7)	1 (1.3)	4 (1.6)		
Family setting					
Monogamous	165 (93.2)	68 (90.7)	233 (92.5)	0.493	0.483
Polygamous	12 (6.8)	7 (9.3)	19 (7.5)		
Ethnicity					
Yoruba	153 (85.0)	69 (90.8)	222 (86.7)	1.555	0.212
Igbo/Hausa	27 (15.0)	7 (9.2)	34 (13.3)		
Religion					
Christianity	111 (61.7)	43 (56.6)	154 (60.2)	0.577	0.447
Islam	69 (38.3)	33 (43.4)	102 (39.8)		
Educational status					
Secondary or lower	39 (21.7)	20 (26.3)	59 (23.0)	0.651	0.420
Tertiary or higher	141 (78.3)	56 (73.7)	197 (77.0)		

### 3.3. Relationship Between Respondents’ Obstetrics and Medical Histories With Knowledge on PC

As indicated in Table 3, of the one hundred and eighty respondents with good knowledge, majority (46.1%) have more than one living children. A significant association exists between respondents’ knowledge of PC and number of living children (*p* = 0.049). Higher proportion (91.5%) of women with no fertility issues had good knowledge of PC (*p* = 0.041). Also, a significant proportion (96.1%) of the respondents without comorbid medical disorders had good knowledge (*p* = 0.039).

**Table 3:** Association between respondents’ obstetrics and medical characteristics with knowledge of permanent contraception (PC).

Variable	PC knowledge		Total (%)	*X* ^2^	*p* value
	Good (%)	Poor (%)			
Number of previous pregnancies					
None	45 (25.0)	16 (21.1)	61 (23.8)	1.427	0.490
1	40 (22.0)	22 (28.9)	62 (24.2)		
> 1	95 (52.8)	38 (50.0)	133 (52.0)		
Number of previous miscarriages					
None	85 (47.2)	44 (57.9)	129 (50.4)	2.801	0.247
1	23 (12.8)	6 (7.9)	29 (11.3)		
> 1	72 (40.0)	26 (34.2)	98 (38.3)		
Ever had pregnancy complications					
Yes	17 (10.1)	8 (11.4)	25 (10.5)	0.090	0.817
No	151 (89.9)	62 (88.6)	213 (89.5)		
Number of previous births					
None	56 (31.1)	16 (21.1)	72 (28.1)	4.301	0.116
1	44 (24.4)	27 (35.5)	71 (27.7)		
> 1	80 (44.4)	33 (43.4)	113 (44.1)		
Number of living children					
None	55 (30.6)	17 (22.4)	72 (28.1)	6.041	0.049
1	42 (23.3)	29 (38.2)	71 (27.7)		
> 1	83 (46.1)	30 (39.5)	113 (44.1)		
Content with sex of children					
Yes	99 (78.6)	47 (75.8)	146 (77.7)	0.183	0.669
No	27 (21.4)	15 (24.2)	42 (22.3)		
Previous fertility issues					
Yes	15 (8.5)	13 (17.3)	28 (11.1)	4.186	0.041
No	162 (91.5)	62 (82.7)	224 (88.9)		
Completed family size after index pregnancy?					
Yes	37 (22.6)	18 (26.9)	55 (23.8)	0.486	0.486
No	127 (77.4)	49 (73.1)	176 (76.2)		
Comorbid medical disorders					
Yes	7 (3.9)	8 (10.5)	15 (5.9)	4.268	0.039
No	173 (96.1)	68 (89.5)	241 (94.1)		

### 3.4. Predictors of Good Knowledge of PC

Respondents with one living child were about twice less likely than those with more than one living child to have good knowledge of PC (AOR = 0.527 and 95% CI = 0.278–0.998). Women who had previous fertility issues were about two times more likely to have good knowledge of PC (AOR = 2.373 and 95% CI = 1.030–5.466) (Table 4).

**Table 4:** Logistic regression analysis of factors associated with the knowledge of PC.

Variable	Odd ratio (OR)	*p* value	95% CI
Number of living children			
None	1.376	0.381	0.674–2.808
1	0.527	0.049	0.278–0.998
> 1 (Ref)			
Previous fertility issues			
Yes	2.373	0.042	1.030–5.466
No (Ref)			
Comorbid medical disorders			
Yes	0.397	0.095	0.134–1.174
No (Ref)			

### 3.5. Relationship Between Respondents’ Characteristics With Perception of PC

Respondents’ perception of PC (*p* < 0.001) and ever had pregnancy complications (*p* = 0.014) were found to be associated with the desire for spousal uptake of PC. There was no significant association between the sociodemographic characteristics status, knowledge of PC, number of living children, children’s gender and desire for spouse to have PC (Table 5).

**Table 5:** Association between respondents’ characteristics and spousal uptake of permanent contraception (PC).

Variable	Spousal PC uptake		Total (%)	*X* ^2^	*p* value
	Yes (%)	No (%)			
Knowledge of PC					
Good	21 (47.7)	65 (58.6)	86 (55.5)	1.471	0.221
Poor	23 (52.3)	46 (41.4)	69 (44.5)		
Perception of PC					
Good	29 (70.7)	9 (9.9)	38 (28.8)	51.043	< 0.001
Poor	12 (29.3)	82 (90.1)	94 (71.2)		
Number of living children					
None	15 (34.1)	31 (27.9)	46 (29.7)	0.573	0.751
1	12 (23.7)	33 (29.7)	45 (29.0)		
> 1	17 (38.6)	47 (42.3)	64 (41.3)		
Ever had pregnancy complications?					
Yes	9 (22.5)	7 (6.5)	16 (10.9)	7.944	0.014
No	31 (77.5)	100 (93.5)	131 (89.1)		
Number of previous miscarriages					
None	25 (56.8)	54 (48.6)	79 (51.0)	1.213	0.540
1	5 (11.4)	11 (9.9)	16 (10.3)		
> 1	14 (31.8)	46 (41.4)	60 (38.7)		
Content with sex of children					
Yes	25 (78.1)	67 (79.8)	92 (79.3)	0.038	0.846
No	7 (21.9)	17 (20.2)	24 (20.7)		
Completed family size after index pregnancy					
Yes	9 (23.1)	25 (24.0)	34 (23.8)	0.014	0.904
No	30 (76.9)	79 (76.0)	109 (76.2)		

## 4. Discussion

Overall, the women studied had high level of awareness and good knowledge of permanent contraception (PC) but most of them are not willing to practice it or allow their spouse to practice it in the future. Despite the availability of modern contraceptive options, Nigeria still faces a serious threat of overpopulation. As the most populous country in sub‐Saharan Africa, with an estimated 210 million people [20], the uptake of modern contraception including PC remains low.

All the women had heard of PC with health workers and media as the foremost sources of information which is consistent with other studies in Nigeria [21, 22]. This shows that the media is a proactive tool in promoting contraception use, thus making it a viable means for information and intervention. Additionally, being antenatal attendees, these women would have had sessions of health education in which contraception is inclusive. In contrast, a study among antenatal attendees in Enugu showed low level of awareness although it assessed only awareness of male form of PC (vasectomy) [23]. The observed good knowledge of PC in our study aligns with the studies by Okunlola et al and Akpor et al which reported a reasonably high knowledge of PC of 66.3% and 94.2% [24, 25] but differs from Tunau et al. study where only 24.1% had good knowledge [22]. This discrepancy might be due to the difference in the level of awareness of PC and the fact that majority of our study population were literate. Higher educational attainment enhances exposure to health information and supports greater autonomy in reproductive rights and health decision.

Despite high awareness, about a quarter of the women are willing to take up PC as a form of contraception in the future after completing their family size. This is similar to findings in previous studies, which reported future uptake of PC ranging from 19% to 29.2% among women in Nigeria, Addis Ababa, and Kenya [21, 26–28]. However, in the Kenyan study, 27.6% of their respondents were willing to consider PC after 3 or less children while in our study, majority were willing to practice PC after 4 or more children. This could be due to the variances in average household size in both countries (Nigeria 5.0 versus Kenya 4.0) [8, 9]. Our study also revealed low future uptake of PC by the women and desire for spousal uptake despite good knowledge, contentment with the sexes of their children, and high literacy levels. This is consistent with national trends of persistently low uptake of 0.2 to 0.4% among women of reproductive age over the past 28 years [8]. Contributing factors may include aversion to surgical procedures, misinformation or inadequate information, and relative cost of PC since all other family planning methods are provided free of charge in Nigeria [8, 29].

Age influenced the willingness to take up PC, with women aged 26–35 being the most likely to consider it. This is consistent with findings from a study in southern Nigeria, where a significant proportion of women aged 26–35 years used family planning methods compared to other age groups [30]. The main reasons for considering PC were the attainment of desired family size and economic reasons which is consistent with the CDC National Vital Statistics [31]. This is not surprising considering the current economic situation in Nigeria with national monthly minimum wage of $31.5 (30,000 naira) which is not enough to raise a family. Other reasons stated for considering PC were doctor’s advice due to multiple births and medical/obstetrics complication(s) associated with deliveries. On the other hand, the most prevalent reasons for declining PC were due to its irreversibility and lack of adequate information about it which substantiates the findings of some studies in Nigeria and Ethiopia [29, 32]. Therefore, it is essential to provide satisfactory information and adequate counselling on PC so as to improve the women′s perception, knowledge and its uptake.

Remarkably, over a quarter of the women will allow their spouse to have PC. This is higher than previous reports from Lagos (13.5%) and Jos (18.75%) in Nigeria, but lower than reported in Port Harcourt (39.0%), possibly reflecting differences in study timing, regional socio‐cultural characteristics, and increasing levels of awareness over time [7, 21, 33]. Women with more than one living child were more knowledgeable about PC. This is expected since PC is usually practiced after attaining desired family size, so there is the possibility that these women have plans of their family size or could have made findings as regards PC. Reasons for declining spousal PC included fear of side effects, religious beliefs, and surgery‐related concerns, consistent with studies in Nigeria and Turkey [7, 26, 34]. Most of the women who support their spouse’s PC uptake have good perception of the procedure which corroborates the report from previous studies [28, 34]. Previous or index pregnancy complications also increased willingness, and this could likely be due to negative experiences and desire to prevent similar outcomes. Though not significant, it is important to note that respondents’ knowledge of PC did not affect their desire for spousal uptake. A similar finding was reported by Akpamu et al. though their study population were illiterate men [35]. The finding that respondents’ spouses will agree to have PC is in keeping with report from Tijani et al. in which the men agree to have PC if only their wives agree or give their support [21]. This confirms that spousal support is an important determinant of contraception uptake.

Our study found no correlation between respondents’ obstetrics characteristics such as number of living children, number of abortions, content with sex of children and completed family size after the index pregnancy with spousal uptake. This is in keeping with the report from Tijani et al study [21].

### 4.1. Strengths and Limitations

This study was limited in that the women who revealed their choice for PC after index pregnancy were not followed up so as to ascertain their decision. Also, this is a hospital‐based study involving predominantly well‐educated women and may limit the generalizability of the findings to the community and uneducated women. In addition, the cross‐sectional design limits the ability to establish causal relationships between the variables studied. Although the use of a self‐administered questionnaire helped reduced interviewer bias, social desirability bias may still have influenced participants’ responses especially given the recent antenatal counselling and the sensitive nature of questions related to PC. However, the relatively low proportion of women willing to adopt PC in the future suggests that social desirability bias was unlikely to have substantial impact. Despite these limitations, the study provided valuable information on the acceptability and intended future uptake of PC among women and their choice for their spouse filling the gaps identified in previous studies [21, 27]. This study has been able to ascertain wives’ desire for spousal uptake and if spouse will agree to use PC. In addition, the perception and knowledge of the women were assessed, which will help in better family planning advocacy regarding PC.

## 5. Conclusion

There is a high level of awareness and good knowledge of PC; however, only a few will practice it or allow their spouse to practice it in the future. Thus, it is essential to consistently promote contraceptive use including PC as well as spousal involvement while counselling and educating women on contraception. The safety and cost effectiveness of PC for both male and female especially for those who have completed their desired family size in our environment should be routinely included in reproductive health education/counselling in light of the high fertility rate, overpopulation and economic situation of Nigeria. Furthermore, the use of multimedia sources to disseminate information on contraception will extend the reach of a family planning and reproductive health issues promotion.

## Conflicts of Interest

The authors declare no conflicts of interest.

## Author Contributions

Oluwasomidoyin Bello and Raymond Takpe conceptualization, design, data collection and analysis, Oluwasomidoyin Bello, Raymond Takpe and Olugbenga Saanu contributed significantly to the draft and final manuscript writing.

All authors have read and approved the final version of the manuscript, [Corresponding Author ‐ Oluwasomidoyin Bello had full access to all of the data in this study and takes complete responsibility for the integrity of the data and the accuracy of the data analysis.

The [lead author‐ Oluwasomidoyin Bello] affirms that this manuscript is an honest, accurate, and transparent account of the study being reported; that no important aspects of the study have been omitted; and that any discrepancies from the study as planned have been explained.

## Funding

The study was self‐sponsored.

## Data Availability

Data will be made available upon reasonable request from the corresponding author.
